# The quality-adjusted life-years in the oncological patients’ health-related quality of life

**DOI:** 10.1038/s41598-022-17942-1

**Published:** 2022-08-09

**Authors:** Karolina Kucnerowicz, Agata Pietrzak, Witold Cholewiński, Piotr Martenka, Andrzej Marszałek, Ewa Burchardt, Erwin Strzesak

**Affiliations:** 1 Medical Services Records Department, Greater Poland Cancer Centre, Garbary 15, 61-866 Poznan, Poland; 2 Electroradiology Department, Poznan University of Medical Sciences, Garbary 15, 61-866 Poznan, Poland; 3 Nuclear Medicine Department, Greater Poland Cancer Centre, Garbary 15, 61-866 Poznan, Poland; 4 Radiotherapy Department, Greater Poland Cancer Centre, Garbary 15, 61-866 Poznan, Poland; 5 Oncologic Pathology and Prophylaxis, Greater Poland Cancer Centre, Poznan University of Medical Sciences, Garbary 15, 61-866 Poznan, Poland

**Keywords:** Gynaecological cancer, Health care, Medical research, Oncology

## Abstract

The oncological treatment can significantly affect patients’ health-related quality of life (HRQoL), which should be monitored to ensure our patients’ well-being. The often-used HRQoL measurer is the quality-adjusted life-year (QALY) indicator of the disease burden, describing both quality and quantity of life lived. The main aim of the study was to discuss the methodology and usefulness of evaluating QALYs using the HRQoL questionnaires: EuroQoL (EQ)-5 dimensions-3 levels (EQ-5D-3L) and EQ visual analogue scale (EQ-VAS) in 32 cervical cancer patients. We obtained the questionnaire and calculated QALYs based on the Gross Domestic Product (GDP) method. In our study, the total scoring of the EQ-Index, EQ-VAS evaluation was 2620 and 2409 points, respectively, which corresponds with the QALYs value of 26.2 and 24.9, respectively. We expressed the QALYs outcome into the economic equivalent of nearly 900,000 US dollars (USD) as the total health profit for both the patients and the healthcare system. Obtaining the QALY factor can help establish the medical management’s influence on the patients’ HRQoL and improve the healthcare services to ensure the best health outcomes.

## Introduction

According to the World Health Organization (WHO), the quality of life (QoL) indicates the individual human perception of the position in the environment, which covers various aspects of self-assessment, including cultural and psychologic aspects^1^. When using the term: health-related quality of life (HRQoL)^2^ measured with quality-adjusted life-years (QALYs), the researched objective is the patient’s self-assessment in terms of general health condition measured with points obtained using specific tests^3–5^. QALYs aim to combine patients’ life length values and their QoL into one index. As a result, the QALY factor expresses the economic benefit of a full year of living in perfect health. The monetary equivalent of the QALY factor establishes the appropriate local lawful regulation.

HRQoL evaluation can be performed numerous times throughout the treatment using standardized and non-standardized methods of analysis. During the study, patients undergo health condition self-assessment using the dedicated survey and the visual analogue scale (EQ-VAS)^6^. The study characterizes general health condition as the ability to normal functioning without the additional help of caregivers. According to the EuroQoL Descriptive System (EQDS) and the National Institute for Health and Care Excellence (NICE) recommendations, the questionnaire used for the HRQoL assessment needs to cover the following criteria: mobility, self-care, usual activities, pain to discomfort ratio, and anxiety to depression ratio. Moreover, each field should be assessed using at least three levels in each dimension (EQ-5D-3L questionnaire). Answers should be then defined as points accordingly to the questionnaires’ scoring. Based on HRQoL tests’ total scoring, we can calculate QALYs. There are various methods of translating the obtained scoring into QALYs. Very often, QALY is expressed as 300% of the Gross Domestic Product (GDP) value^7,8^. Moreover, QALYs can be combined with several costs of medical procedures and expressed as a final common denominator of procedures’ cost and outcomes^9^. Depending on the evaluation method, QALYs can be used, i.e., to compare the impact of various therapeutic methods on patients’ HRQoL and adjust patients’ management accordingly with its cost-effectiveness^9^.

HRQoL measured with QALYs help to represent the patient's well-being status in a widely understood, measurable, and reproducible manner. It is also a universal economic indicator that can be applied to all patients and diagnoses in any country and uses every single currency available. In this study, we mentioned the widely used currency of US dollars as an example of the QALY measurement outcome.

Although WHO developed the term QoL in 1994, the original studies comparing the EQ-5D-3L questionnaire database and the EQ-VAS evaluation have not been widely investigated^10,11^. Moreover, the QALY factor evaluation has not been widely exercised in cervical cancer patients. The study aimed to discuss the methodology and purposes of evaluating QALY factor based on the results of the standardized and non-standardized HRQoL tests performed in a group of the oncological patients treated in our institution, as well as, to establish whether it is useful in evaluating our patients’ well-being.

## Results

### EQ-Index and EQ-VAS evaluation

In this retrospective study, we analyzed the HRQoL of 32 histopathologically examined cervical cancer patients, using the QALY factor evaluation with the EQ-5D-3L questionnaire and the EQ-VAS assessment.

Table 1 shows the results obtained in the examined group.

**Table 1:** The cervical patients HRQoL evaluation: EQ-Index with EQ-5D-3L versus EQ-VAS (*source*: original data).

I.n.^a^	Age (years)^b^	Diagnosis duration (years)^c^	EQ-Index^d^	EQ-VAS^e^
1	60	2	30	20
2	60	2	90	90
3	60	1	90	85
4	**60**	**2**	**100**	**90**
5	60	1	70	90
6	**60**	**2**	**100**	**100**
7	60	2	60	50
8	**61**	**2**	**70**	**100**
9	61	2	90	90
10	61	2	90	65
11	61	2	90	89
12	61	2	80	60
13	61	2	80	70
14	62	1	80	40
15	62	2	50	70
16	62	2	90	50
17	**63**	**2**	**100**	**60**
18	**63**	**2**	**100**	**80**
19	**65**	**2**	**100**	**85**
20	**65**	**2**	**100**	**100**
21	65	1	90	80
22	66	2	70	90
23	**66**	**1**	**100**	**100**
24	66	1	90	80
25	66	2	80	70
26	66	2	70	90
27	67	2	90	80
28	67	2	60	40
29	**67**	**1**	**100**	**80**
30	68	1	90	90
31	68	2	90	90
32	69	2	30	35

*HRQoL* health-related quality of life, *EQ* EuroQoL system, *5D* 5 dimensions, *3L* 3 levels of assessment, *VAS* visual analogue scale, ^a^initial number, ^b^in 2017, ^c^time from diagnosis, ^d^questionnaire results, ^e^patients’ subjective evaluation, bold font: the highest EQ-Index level observed.

Most often, the results in both datasets were comparable, especially in patients with a low HRQoL level. The total scoring obtained with the EQ-Index and the non-standardized self-assessment (EQ-VAS) evaluation were: 2620 and 2409 points, respectively.

Table 2 shows the specific measurements, obtained in this study, including the diagnosis duration, EQ-Index, and EQ-VAS evaluation.

**Table 2:** The measurements (*source*: original data).

Characteristics	Diagnosis duration (years)	EQ-Index	EQ-VAS
Mean ± S.D.	1.8 ± 0.4	82 ± 19	75 ± 21
Range	1.0–2.0	30–100	20–100
CI_95_	[1.6; 1.9]	[75; 89]	[68; 83]
Median	2.0	90	80
Mode	2.0	90	90

*EQ* EuroQoL, *VAS* visual analogue scale, *S.D.* standard deviation, *CI*_*95*_ confidence interval (valid for 95% of examined population).

Due to the Shapiro–Wilk test’s results, the EQ-Index, EQ-VAS distributions differed significantly from Gaussian with *P* of < 0.001, 0.003, respectively. We compared the EQ-Index, EQ-VAS evaluation results. The Wilcoxon’s pair test for dependent variables showed that the differences between standardized scoring and the patients’ subjective evaluation were statistically insignificant, with *P* = 0.06. In 8 of the examined patients, we observed the maximal EQ-Index scoring; in 3, EQ-Index and EQ-VAS were equal. In most of the studied cases, the diagnosis duration approximated at 2 years (24 from 32 patients). Therefore, we could not perform reliable diagnosis duration—HRQoL scoring statistical correlation analysis.

### The QALY factor evaluation

In our study, the total EQ-Index and the EQ-VAS scoring collected by the examined patients were: 2620 points, and 2409 points, respectively. Thus, the total QALYs obtained in this group were 26.2 and 24.9 QALYs. The most recent PLN and USD exchange rate approximates 5:1, and the GDP is 55,586 PLN^8^. Thus, the QALY as 300% GDP equalled 166,758 PLN^8^. When considering the most recent PLN to the USD exchange rate (5:1), 1 QALY unit worth can be concluded to equal approximately 33,352 USD. Based on the EQ-Index and EQ-VAS evaluation, the total QALYs obtained in this study were 873,822 USD and 830,465 USD, respectively. QALYs are considered the indicators of mutual patient and the healthcare system health-benefit. It is then safe to conclude that our patients and the institution gained an economic health profit of nearly 900,000 USD. QALYs can be successfully used to compare the impact of the different therapeutic approaches on our patients’ HRQoL in an easy, convenient manner.

We analyzed the economic equivalent of the obtained EQ-Index and EQ-VAS using GDP method for QALYs calculation. In this example of QALYs economic outcomes, we used the USD currency only.

Based on our analysis, the mean ± S.D. QALY factor obtained with the EQ-5D-3L (EQ-Index) and EQ-VAS was 0.8 ± 0.2 (range: 0.3–1.0, CI_95_ [0.7; 0.9], median 0.9, mode 0.9) and 0.8 ± 0.2 (range: 0.2–0.1, CI_95_ [0.7; 0.8], median 0.8, mode 0.9).

Considering the GDP method of recalculation and the PLN to USD exchange rate, the mean ± S.D. EQ-Index economic equivalent was 27,307 ± 6364 USD (range: 10,006–33,352 USD, CI_95_ [25,012; 29,601 USD], median 30,017 USD, mode 30,017 USD). The mean ± S.D. EQ-VAS equivalent was 25,108 ± 7029 USD (range: 6670–33,352 USD, CI_95_ [22,573; 27,642 USD], median 26,682 USD, mode 30, 017 USD).

## Discussion

The oncological treatment aims to cure the disease, obtain the best possible patient clinical outcomes, and ensure a high HRQoL of treated patients during and after the therapy. However, oncological patients’ management is challenging considering the clinical and economic aspects of the diagnosis and treatment. The diagnostic and therapeutic procedures need to be considered and planned due to illness characteristics, patients’ individual needs, and the allocation of the financial resources in the medical institution.

The most reliable health indicator is the patient’s self-assessment. Following the WHO definition of health, the patients’ physical and mental health must be considered equally important. Remaining in good health allows the patient to provide an active social life, work and be more self-sustainable^12^. Due to its complexity, the patient’s health condition needs to be evaluated using a precise, unbiased testing tool. Moreover, the assessment method must be short, understandable for the patient, and easy to interpret by the investigator. Therefore, the routinely performed, standardized HRQoL evaluation covering various characteristics is vital for successful oncological management^1,12^.

According to the literature^13,14^, the cancerous disease occurrence can be considered age-related, affecting the HRQoL scoring after the therapy. The incidence of malignant neoplasms increases with age, which can be observed among, i.e. head and neck, lung, prostate or gynaecological cancer patients. However, some authors^14^ suggest that the likelihood of cancer occurrence should be distinguished from the ageing process as natural and not necessarily predisposing to the illness.

In this study, we have evaluated the HRQoL-dedicated factors in one group of elderly patients. According to the literature^15–17^, elderly cancer patients show lower QALY factor values and indicate decreased EQ-VAS when compared to younger patients. Elderly patients over 60 years old (y.o.) are considered a high-risk group for post-therapeutic or health-related anxiety and depression. Elderly patients with various disabilities often need support in their daily routine due to limited mobility, anxiety, depression, and overall fatigue. At the same time, it is the most extensive group among the oncological patients. Therefore, this group seems adequate for the study regarding the HRQoL analysis.

In this study, we analyzed HRQoL and QALYs within the group of patients diagnosed with cervical cancer as a challenging disease for women. Cervical cancer has been reported to be 4th most commonly occurring malignancy in women worldwide and 6th in Poland^18–21^. According to the literature^18–21^, the incidence of cervical cancer is the highest in Eastern Africa and Central and Eastern Europe and the lowest in Western Asia compared to the worldwide cervical cancer morbidity. The age distribution of cervical cancer patients seems comprehensive and includes the high incidence among young and elderly patients. However, based on the local National Cancer Registry data, the highest incidence of cervical cancer in Poland has been observed in patients over 60 y.o.^21^.

In this study, we examined the HRQoL of the 32 cervical patients diagnosed and treated in our institution. We evaluated the group well-balanced in terms of age, and the therapeutic protocol applied to our patients to obtain the most reliable results. The age group was selected based on cervical cancer epidemiology^21^.

We analyzed our patients’ HRQoL using the EQ-Index obtained with the standardized EQ-5D-3L questionnaire and the non-standardized EQ-VAS test. We observed the highest scoring in 8 EQ-Index and in 4 EQ-VAS datasets. In 3 of the studied patients, the EQ-Index equalled the EQ-VAS. The mean EQ-Index and EQ-VAS factors values were 82, 75 points, respectively, and the most often reported EQ-Index, EQ-VAS values (mode) were 90, 80 points, respectively. In this study, we found no statistically significant differences between the standardized evaluation and the self-assessment scoring, showing that both tools can help measure patients’ HRQoL. In this sample, the standardized questionnaire was not superior to the EQ-VAS test in evaluating HRQoL of the cervical cancer patients, which confirms that the patients can precisely indicate their health concerns and their impact on daily routine. A relatively high score may also suggest that the treatment protocol applied to our patients does not overly affect their HRQoL.

Based on the HRQoL assessment and the obtained scoring, we calculated the QALYs to present the benefits of a high QoL using the economic indicator. We followed current official guidelines describing QALY as 300% of national GDP and recalculated the local currency of the polish zloty (PLN) to widely used US dollars (USD)^7,8,22^. In this group, the total EQ-Index and the EQ-VAS scoring were: 2620 and 2409 points, respectively, which equals 26.2 and 24.9 QALYs, respectively. The total economic benefit of the obtained QALYs was 873,822 and 830,465 USD, respectively. QALY has been considered a benefit for both the patient and the healthcare system and a useful procedural cost-effectiveness measure. Comparing the total benefits and the oncological patients’ management costs can be used to choose the best clinical pathway and evaluate the future financial resources allocation according to the institution and society’s health-related needs. Such an economically adjusted clinical approach is used for the financial resources’ placement plan in many medical institutions to ensure that all the health-related needs of the society have been covered.

The study aimed to discuss the methodology and purposes of evaluating the QALY factor based on the results of the standardized and non-standardized HRQoL tests performed in a group of oncological patients treated in our institution to establish the most accurate way to evaluate our patients’ well-being. We attempted to conclude whether we can assess the impact of the treatment applied to our patients on their HRQoL based on our research. The main limitation of this preliminary study is a small study sample and the lack of the control group and recurrent cervical patients’ inclusion. Comparing the HRQoL of the primary and recurrent patients seems inadequate as the QoL also depends on the number of procedures and their invasiveness. The study demands expanding the studied group, providing the appropriate control dataset, and comparing the obtained results between the groups of different diagnoses. Based on this sample, we cannot conclude the most appropriate method of evaluating HRQoL with complete certainty. Thus, expanding the database is needed.

QALYs help to assess the cost-effectiveness of the patients’ management and to justify or adjust the therapeutic procedures applied to the patient in the medical institution. In conclusion, it helps to choose what is best for our patients to maintain a healthy life after the procedure by evaluating the impact of clinical pathways on their HRQoL. The HRQoL evaluation using the QALY factor measurement might help to plan the allocation of the medical institution's financial resources. Accordingly, with the goals of the HRQoL assessment, groups of diagnosis in which the total QALY factor are the lowest should be additionally financially supported. The resource allocation should be preceded, i.e. by a comparative analysis of the standardized questionnaire and the patients’ self-assessment scoring to establish which elements of the oncological management should be improved. A high HRQoL indicates the patients’ well-being. A healthy patient is a person who can actively participate in social life and benefits society with their personal development. However, for this to happen, patients should be provided with the highest possible healthcare and satisfying post-therapeutic QoL. Thus, providing appropriate diagnostic and therapeutic management in conjunction with ensuring a high HRQoL should be considered equally relevant healthcare goals.

## Methods

### Bioethics

The paper is based on the retrospective analysis approved by the Local Bioethical Committee (Poznan University of Medical Sciences, prof. Pawel Checinski, date of approval: 16.01.2020). This study includes original, retrospectively analyzed, unsponsored, single-institutional studies, performed in the year 2017 upon the generous gift of the User Support Officer's EuroQol Research Foundation, available online: www.euroqol.org. The study was carried out in accordance with relevant guidelines and regulations. Informed consent was obtained from all the participants involved in the study.

### Database

Gynecological cancer patients are especially vulnerable due to several side-effects that therapeutic methods applied to them carry. Those side-effects and post-therapeutic complications may significantly decrease women self-assessment, and, therefore, their HRQoL. In this retrospective study, we have analyzed the HRQoL of 32 histopathologically examined cervical cancer patients, using the QALY factor evaluation based on the EQ-5D-3L questionnaire and the EQ-VAS assessment. The studies have been carried out in the Greater Poland Cancer Centre, Poznan, Poland in 2017. According to data collected within the last five years, our hospital treats nearly 15% of cervical cancer patients in the region and approximately 2% countrywide.

The examined group was well balanced in terms of age and consistent with the epidemiology of cervical cancer in Poland^21^. In this group of cervical cancer patients, the mean age was 63 ± 3 y.o (range: 60–69 y.o.). The median was 63 y.o. and the mode was 60 y.o. All studied subjects have been examined using the questionnaire EQ-5D-3L and EQ-VAS assessment within one (range: 11–13 months) to two years (range: 19–24 months) after establishing the diagnosis (diagnosis duration). All patients underwent comparable therapeutic pathways, including surgery and external beam radiotherapy. In each patient, we performed HRQoL evaluation for approximately 6 months since the last therapeutic procedure.

To provide all necessary background data, we used the National Center for Biotechnology Information (NCBI; United States of America National Library of Medicine, 8600 Rockville Pike, Bethesda MD, 20,894 USA, including Medline, Pubmed, Pubmed Central, and other scientific databases) literature resources published within 1997–2021. We followed the searching criteria: age-related QoL, cervical cancer; EQ-5D-3L; EQ-VAS; HRQoL; oncology; QALY; QoL; quality of life. We narrowed down the research outcome to the EQ-5D-3L questionnaire as used in this study accordingly with NICE recommendation^2^. We followed the official Polish Central Statistical Office published via Agency for Health Technology Assessment and Tariff System (AOTMiT), and Polish National Cancer Registry reports to present QALYs calculation methodology and cervical cancer epidemiology^7,8,21^.

### Study performance

Completing the EQ-5D-3L questionnaire and the patient’s EQ-VAS self-assessment were conducted in a study room, ensuring intimacy and comfort during the examination. The medical assistant qualified in the study methodology provided each patient with detailed instructions for filling in questionnaires. The assistant remained nearby at the disposal of the respondent in case they needed any support in answering questions.

The assistant did not influence the study and did not suggest the answers. After completing the questionnaires, the medical assistant discussed with the patient the overall experience of the study. For most of the studied patients, the most difficult seemed to be evaluating their mental health and overall well-being (EQ-VAS).

### The standardized questionnaire construction and QALYs evaluation method

The standardized questionnaire EQ-5D-3L (Table 3) scoring includes five questions with three different answers with one possible option to choose from by the respondent.

**Table 3:** The EQ-5D-3L questionnaire’s structure (*source*: ^23^).

EQ-5D-3L questionnaire structure		
Characteristic	Answers (one to choose from below mentioned)	Scores
Mobility	I have no problems walking about	20
	I have some problems walking about	10
	I am confined to bed	0
Self-care	I have no problems with self-care	20
	I have some problems washing or dressing myself	10
	I am unable to wash or dress myself	0
Usual activities	I have no problems with performing my usual activities	20
	I have some problems with performing my usual activities	10
	I am unable to perform my usual activities	0
Discomfort and pain	I have no pain or discomfort	20
	I have moderate pain or discomfort	10
	I have extreme pain or discomfort	0
Anxiety and depression	I am not anxious or depressed	20
	I am moderately anxious or depressed	10
	I am extremely anxious or depressed	0

Scoring: 0–100 points, 0—worst possible state of health, 100—best possible state of health; *EQ* EuroQoL system, *5D *5 dimensions, *3L* 3 levels of assessment.

The available questionnaire’s scoring equals 100 points, which can be considered the one health-adjusted year of life of the evaluated patient—1 QALY unit. The QALY unit can be calculated using various methods. One of the most used pathways is expressing QALY as a GDP derivative, specifically: 3 × GDP. Each country's GDP tends to change over time. Thus, the total QALY value varies as well^7,8^. In this study, we presented QALYs calculation considering current local GDP and PLN versus USD approximated ratio as 5:1, respectively^22^. We recalculated PLN to USD to present the study outcome using the widely used currency aside from local. It is worth mentioning that the evaluation outcome depends on the currency exchange rate and can be applied to any local currency^7–9^.

In this group, we divided the total number of points by the maximal scoring of 100 points. We expressed it as the 300% GDP to establish the total economic benefit of the obtained HRQoL among the cervical cancer patients who underwent oncological treatment.

In this study, we obtained QALYs based on the questionnaire scoring (EQ-Index and EQ-VAS derivatives)^7,8,13^, where:

$$\begin{aligned} {1}\;{\text{QALY}}\;{\text{unit}} & = {1}00\;{\text{points}}\;\left( {{\text{questionnaire}}\;{\text{total}}\;{\text{scoring}}} \right), \\ {\text{QALYs}} & = \left( {{\text{obtained}}\;{\text{scoring}}/{1}00} \right) \\ \end{aligned}$$

When establishing the economic equivalent, we followed the methodology based on GDP:

$${1}\;{\text{QALY}} = {3}00\% \;{\text{GDP}}.$$

### Statistical evaluation

In this study, we used the statistical significance level of 5% (confidence interval at the level of 95%, CI_95_), and we obtained the statistical tests’ results, considering the *P* value. We followed a null and alternative hypothesis (H_0_, H_a_, respectively) assumptions: H_0_ suggested the variables’ distribution normality or that the obtained differences between the calculations insignificance (*P* > 0.05), Ha—true distribution significantly differed from Gaussian, and that the observed differences were significant (*P* < 0.05). Due to sample-size (less than 1000 cases), we followed the Shapiro–Wilk test’s results to establish the following variables distribution: EQ-Index, EQ-VAS. Accordingly with the Shapiro–Wilk test’s results, we used the appropriate test to compare EQ-Index and EQ-VAS as obtained in same patients, dependent variables. QALYs presented and obtained in this study are EQ-Index and EQ-VAS derivatives. Thus, comparing QALYs obtained with above-mentioned factors have been omitted.

We used the STATISTICA, StatSoft software, version 13.3 (TIBCO Software, Palo Alto, California, USA, available upon individual license).

## Data Availability

The datasets analyzed during the current study has been included in the paper’s content.
