# Author Correction: The quality-adjusted life-years in the oncological patients’ health-related quality of life

**DOI:** 10.1038/s41598-022-18893-3

**Published:** 2022-08-29

**Authors:** Karolina Kucnerowicz, Agata Pietrzak, Witold Cholewiński, Piotr Martenka, Andrzej Marszałek, Ewa Burchardt, Erwin Strzesak

**Affiliations:** 1 Medical Services Records Department, Greater Poland Cancer Centre, Garbary 15, 61-866 Poznan, Poland; 2 Electroradiology Department, Poznan University of Medical Sciences, Garbary 15, 61-866 Poznan, Poland; 3 Nuclear Medicine Department, Greater Poland Cancer Centre, Garbary 15, 61-866 Poznan, Poland; 4 Radiotherapy Department, Greater Poland Cancer Centre, Garbary 15, 61-866 Poznan, Poland; 5 Oncologic Pathology and Prophylaxis, Greater Poland Cancer Centre, Poznan University of Medical Sciences, Garbary 15, 61-866 Poznan, Poland

Correction to: *Scientific Reports* 10.1038/s41598-022-17942-1, published online 09 August 2022

The Funding section in the original version of this Article was omitted. The Funding section now reads:

“Funded by the Greater Poland Cancer Centre scientific grant no 7/02/2020/DOIN/WCO/002.”

The original Article has been corrected.

